# Supplementary material for: The interactome of intact mitochondria by cross-linking mass spectrometry provides evidence for coexisting respiratory supercomplexes
Source: Mol Cell Proteomics. 2017 Dec 8;17(2):216–32. doi: 10.1074/mcp.RA117.000470 (PMC5795388; doi:10.1074/mcp.RA117.000470)
Supplement: Supplemental Data [file supp_RA117.000470_133922_0_supp_23978_fzffwf.zip › spectra_annotation/mito_spectra_annotation/290-1-2-1-4-1.pdf]

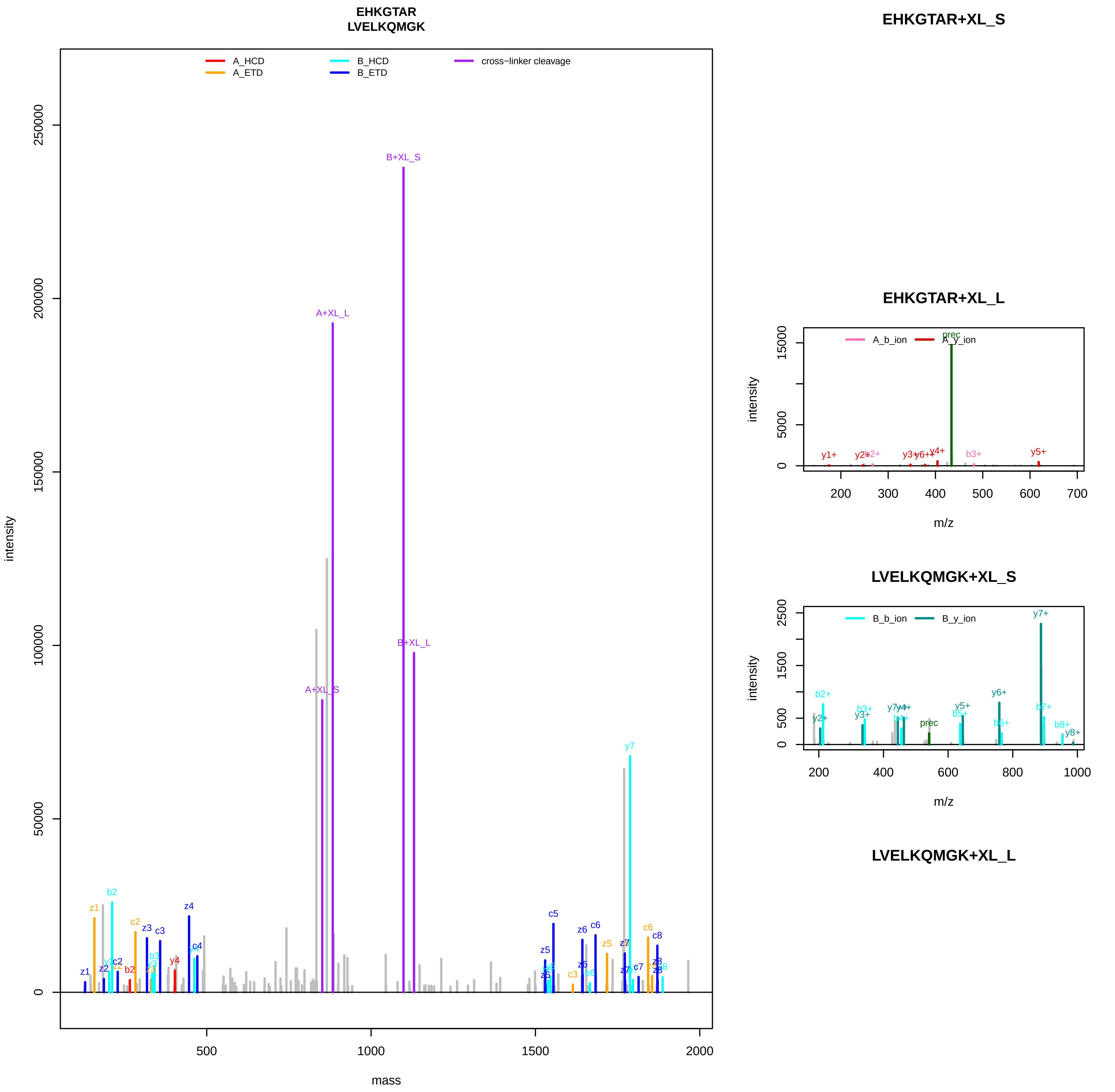
EHKGTAR
LVELKQMGK
A_HCD
A_ETD
B_HCD
B_ETD
cross−linker cleavage
B+XL_S
A+XL_L
B+XL_L
A+XL_S
y7
z1
b2
c2
z3
c3
z4
c4
y4
z5
c5
z6
c6
z5
c6
c8
intensity
mass
0
50000
100000
150000
200000
250000
500
1000
1500
2000
EHKGTAR+XL_S
EHKGTAR+XL_L
A_b_ion
A_y_ion
prec
y1+
y2+
b2+
y3+
y6+
y4+
b3+
y5+
intensity
m/z
0
5000
15000
200
300
400
500
600
700
LVELKQMGK+XL_S
B_b_ion
B_y_ion
y7+
b2+
y2+
b3+
y3+
y7y4+
b4+
prec
y5+
b5+
y6+
b6+
b7+
b8+
y8+
intensity
m/z
0
500
1500
2500
200
400
600
800
1000
LVELKQMGK+XL_L
